# Prevalence of post-traumatic stress disorder and associated factors among police officers in Central Gondar Zone, North West Ethiopia, 2023: institutional-based cross-sectional study

**DOI:** 10.3389/fpsyt.2024.1338833

**Published:** 2024-09-03

**Authors:** Biruk Adugna, Bizuneh Tesfaye, Dawed Ali, Yohannes Mirkena, Wondale Getinet

**Affiliations:** Department of Psychiatry, College of Medicine and Health Science, University of Gondar, Gondar, Ethiopia

**Keywords:** prevalence, PTSD, police officer, trauma, Ethiopia

## Abstract

**Background:**

Post-traumatic stress disorder (PTSD) is a psychiatric disorder that follows exposure to a traumatic or stressful life event. Police officers are exposed to a number of traumatic events that put them at risk of developing post-traumatic stress disorder. Previous studies have found the prevalence of post-traumatic stress disorder among police officers to be varied and inclusive. However, in Ethiopia, little is known about the prevalence of post-traumatic stress disorder and associated factors among police officers. Therefore, assessing the prevalence and associated factors of post-traumatic stress disorder among police officers might have a plausible role in future investigations.

**Objective:**

The aim of this study was to assess the prevalence of post-traumatic stress disorder and associated factors among police officers in Central Gondar Zone, North West Ethiopia, 2023.

**Methods:**

An institutional-based cross-sectional study was conducted in Central Gondar Zone by using self-administered and semi-structured questionnaires. A multi-stage cluster sampling technique was employed to select a sample of 634 participants. Post-traumatic stress disorder was assessed using the PTSD Checklist DSM-5. The collected data were coded and entered using Epi data Software version 4.6.02 and then exported to STATA version 14 for analysis. Bivariable and multivariable logistic regression analyses were done to identify factors associated with post-traumatic stress disorder. Statistically significant results were declared at a 95% confidence interval (CI) of a *p*-value less than 0.05.

**Results:**

The prevalence of post-traumatic stress disorder was found to be 15.2%, with a 95% CI of 12.5% to 18.3%. In the multivariate analysis, female (AOR = 3.36, 95% CI 1.95–5.78), being directly exposed to traumatic events (AOR = 2.01, 95% CI 1.16–3.48), current alcohol use (AOR = 2.90, 95% CI 1.65–5.12), and having poor social support (AOR = 4.25, 95% CI 1.58–11.36) were factors significantly associated with post-traumatic stress disorder.

**Conclusion:**

According to this study, police officers suffered from a significant burden of post-traumatic stress disorder. Female sex, police personnel directly exposed to traumatic events, current alcohol users, and those who had poor social support were found to be strongly associated with post-traumatic stress disorder. Therefore, early detection and intervention are crucial to mitigating the overall problem.

## Introduction

According to the DSM-5, post-traumatic stress disorder is a psychiatric disorder following one or more traumatic or stressful life events primarily characterized by intrusion, avoidance, and mood changes as well as cognition and hyper-vigilance lasting for more than a month (1). It is also a chronic, under-recognized, and under-treated psychiatric consequence of trauma (2).

Police officers are a group that is particularly vulnerable to traumatic stressors as defined in the DSM-5 criteria “A” for PTSD, where they may encounter at least three traumatic events every 6 months of service (3), which include seeing and handling the body of the deceased, dealing with sexually harassed victims, encountering violence, witnessing serious traffic accidents, suicide, and being involved in shootings. As such, they develop PTSD (4).

The World Health Organization (WHO) global disease burden survey predicts that mental illness, including stress-related disorders, will be the second leading cause of disability by the year 2023 (2). Every year, approximately eight million individuals worldwide suffer from PTSD (5). It is believed that 77% of PTSD sufferers in low- and middle-income countries remain untreated (6). A total of 60 cross-sectional studies and seven longitudinal studies, including 272,463 police officers from 24 different nations, found a 14.6% overall pooled point prevalence of PTSD (7). A systematic review has shown that the prevalence of PTSD in police officers varied significantly from 0% to 44% (8). A brief online survey conducted among 1,355 active-duty officers from across the United States found that 47% of the sample screened positive for PTSD, which is approximately nine to 10 times greater than the prevalence seen in the general population (9). On average, police officers reported 19.5 different types of traumatic events. As a result, female officers experienced more PTSD symptoms than their male colleagues (10). Approximately 15% of American police personnel suffer from PTSD (4). Another cross-sectional study conducted in South Africa among 392 field police officers reported a 7.4% prevalence of PTSD (11).

There were different factors that affected PTSD. These include the presence of psychiatric comorbidities, alcohol use disorders, a low educational level, a lack of training, low social support, and trauma exposure (11, 12). The risk of PTSD is also higher among police officers who are female and young (10, 11, 13, 14). However, due to the fact that police officers are expected to be effective decision-makers and independent problem solvers while working with the system (15), such factors can harm the officers’ mental health and hinder their capacity to carry out their duties to the public. In addition, behavioral dysfunction such as substance misuse, aggressiveness, and suicide may result from the long-term impacts of PTSD on police officers (4). Police officers with PTSD also tend to have faced different problems, such as poor performance and interaction with citizens, an inability to effectively carry out their duties, and impaired social interactions with their loved ones, friends, colleagues, and even the public, experiencing mood swings or aggressive or violent behavior (16, 17).

There has been a lot of research on PTSD in society as a whole, but less on the impact of PTSD on police officers (16). Despite the fact that police officers are at a high risk of developing psychiatric disorders, previous studies have received more attention in western countries (11), although previous studies would suggest that a very real problem exists and that police officers are at a high risk of developing mental health disorders such as PTSD.

Therefore, this study was conducted to fill the gap and determine the magnitude and associated factors of PTSD among police officers, which could facilitate an early and evidence-based intervention to mitigate further problems associated with PTSD. There is a deficit of research carried out in Ethiopia, so the extent and scope of the problem is not known. Therefore, this study was conducted to determine the magnitude and associated factors of PTSD among police officers, which could facilitate early intervention to mitigate further problems associated with PTSD.

Furthermore, the current study aimed to give important recommendations for scientific communities to reduce the risk factors of post-traumatic stress disorder and provide information for policymakers and health professionals to design appropriate solutions for the problem and develop prevention and treatment strategies.

## Materials and methods

The study was conducted in the Central Gondar Zone, Amhara Region, Ethiopia. It is found in the northwest part of Ethiopia. Gondar, which served as Ethiopia’s capital during the Gondarian era and is currently the administrative center of the Central Gondar Zone, is a city with a mean elevation of 2,133 meters above sea level (m.a.s.l.) and is located at 12°36′ N latitude and 37°28′ E longitude. The total population residing in Central Gondar Zone was 2,896,928 (18); Central Gondar Zone City Administration Police Department has 20 police stations, which consist of 1,350 police officers. According to Cambridge English, a police officer is referred to as a male or female member of the police force. Among those, 1,155 are male and 195 are female police officers. A police station is a building that serves to accommodate police officers and other members of staff.

### Sample size and sample procedure

**Figure 1:**
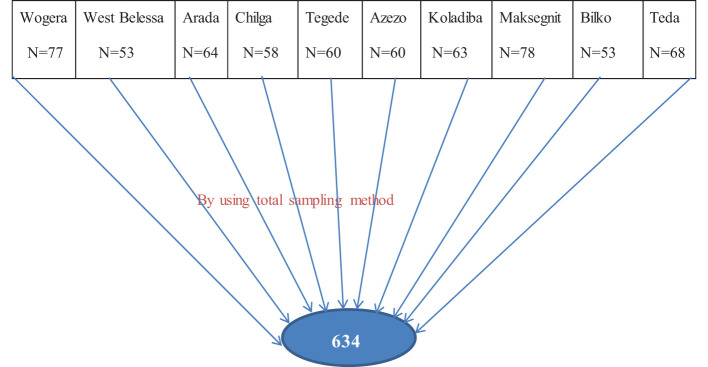
Sampling technique and procedure for the prevalence of PTSD and associated factors among police officers in Central Gondar Zone, North West, Ethiopia.

An institutional-based cross-sectional study was conducted from February 24 to March 22 2023. A multi-stage cluster sampling technique was employed to select the study participants. There are 20 police stations in Central Gondar Zone, with a total of 1,350 police officers. At stage one, the 10 police stations (clusters) were selected randomly using the lottery method. Then, data was collected from every unit within the selected clusters in one stage. At stage two, all police officers who were presented at work during data collection time were included to this study by using the total sampling method, while police officers who were in serious conditions like acute illness during data collection time were excluded. A single population proportion formula with an assumption of a 95% confidence level, a 5% margin of error, and prevalence *p* = 50% because no published similar study is available on the police population in Ethiopia, with 95% confidence interval, 5% margin of error, and non-response rate = 10%. Then, the final calculated sample size was 634 (see **Figure 1**).

### Schematic representation of sampling technique

Here there is which shows the schematic presentation of sampling technique (I can share the figure again and it should be put here.

### Study variables

The dependent variable was PTSD and measured as a dichotomous variable (yes/no). Independent variables included socio-demographic factors—age, sex, police rank, marital status, educational status year of service, and living arrangements; clinical variables include a previous history of mental illness, a family history of mental illness, chronic medical condition, and a history of suicidal ideation and attempt; substance-related factors were ever/current alcohol consumption, cigarette smoking, and khat chewing; and psychosocial factors were poor social support and level of exposure.

### Operational definitions

Post-traumatic stress disorder: Post-traumatic stress disorder was measured by using a 20-item post-traumatic checklist (PCL-5). Individuals who scored greater/equal to 33 were considered as having (probable) post-traumatic stress disorder (19).

Ever use of substance: using at least one of any specific substance for non-medical purposes at least once in your life (alcohol, khat, and cigarette) (20).

Current substance use: Those who have used substances in the last 3 months (21).

Mental illness: Have you ever been faced with any known mental illness? If they say yes, it was considered as having mental illness.

Chronic medical illness: Have you ever been faced with any known chronic medical illness? If they say yes, it was considered as having chronic medical illness.

Family history of mental illness: Do you have a family member who has ever known mental illness? If the answer is “yes”, the respondent has a family history of mental illness.

History of suicidal ideation and attempt: Have you ever thought of killing and made an attempt to kill yourself? If the answer is “yes”, the respondent has a history of suicidal ideation and attempt.

Social support: From the Oslo-3 Social Support Scale, police officers who scored 3–8 have poor social support, 9–11 have moderate social support, and 12–14 have strong social support (22).

Level of traumatic exposure: It was assessed by using structured yes/no questions to identify traumatic experiences involving actual or threatened death, war, serious injury, homicide or suicide, and sexual violence. It could be something that happened to you directly or something you witnessed while others were brutally harassed or killed.

### Data collection tools and materials

A structured questionnaire was developed in the English language, which is adapted from different literature and has five parts. The questionnaire was translated into the local language.

Socio-demographic characteristics such as age, sex, educational level, and the like were collected by using structured socio-demographic questionnaires.

An outcome variable, the prevalence of PTSD, was assessed using the Posttraumatic Checklist (PCL-5), which contains 20 items with scores ranging from 0 to 80, representing four subscales: re-experience (intrusion), avoidance, negative changes in cognition or mood, and an increase in arousal or reactivity. The answers are marked on a five-point scale, where 0 means not at all and 4 means very strong. We had adapted the PCL-5 in which the validity and reliability of the tool have been tested and proven on general populations with a Cronbach’s alpha value of 0.96 for the overall PCL-5 result (19).

Substance-related factors, which comprise of substance use for its assessment, which is current use and ever use, were adapted from the ASSIST (Alcohol, Smoking, and Substance Involvement Screening Test), a well-validated instrument developed by the World Health Organization (20, 21).

Clinical factors, such as history of mental illnesses, chronic medical illness, family history of mental illness, and suicidal ideation and attempt, were assessed by using structured yes/no questions.

Social support: The Oslo three-item social support scale was used to measure the strength of social support. The score that ranges between 3 and 8 is classified as “poor social support,” a score between 9 and 11 as “intermediate social support,” and a score between 12 and 14 as “strong social support” (23). This tool is validated for this research and has been validated in our country (in Ethiopia context). Finally, the level of traumatic exposure was assessed by using structured yes/no questions.

### Data collection

Data were collected using a self-administered approach (the participants filled out the questionnaires by themselves). Four professional nurses were involved (the role of those nurses were only just facilitating and enumerating during data collection), and two psychiatry profession supervisors and the principal investigator participated. For those data collectors and the supervisor, 1-day training was given before the actual data collection date. During the training, the objective of the study, the data collection methods and tools, and how to handle ethical issues were briefly discussed with the data collectors. The structured questionnaire was also profoundly discussed by going through each question with clarification for doubt.

### Data quality control

To identify potential problems with the data collection tools, the questionnaire was translated appropriately into the local Amharic language to be understandable by all participants. The questionnaire was pretested 1 week prior to the actual data collection time on 5% of the sample (*n* = 32) at Dabat Woreda police station. Therefore, the dependent variable tool assessment DSM-5 (PCL-5) Cronbach alpha was 0.969. Each completed questionnaire was checked. The collected data was properly handled, reviewed, and checked for completeness and consistency by the supervisor and principal investigator each day.

### Data processing and analysis

The collected data were coded, edited, checked, and entered into Epi data Software version 4.6.02 and then exported to STATA version 14 for analysis. The socio-demographic characteristics and other factors of the respondents were analyzed by descriptive statistics (percentage, mean, and standard deviations). Bivariable and multivariable logistic regressions were used to identify the independent predictors of PTSD. Then, variables with *p*-value <0.2 on bivariable analysis were entered into multivariable logistic regression. Then, a *p*-value <0.05 was considered statistically significant, and the strength of associations was determined by using the adjusted odds’ ratio (AOR) at 95% CI. The Hosmer and Lemeshow test was performed to test the logistic regression model for goodness of fit (*P* = 0.82). The variables were tested for multicollinearity (VIF = 0.75).

### Ethical consideration

All procedures undertaken during the time of data collection were in accordance with the ethical review board of the University of Gondar and the Declaration of Helsinki. A formal letter of permission was obtained from the Department of Psychiatry. Written informed consent was obtained from the respondents; the right to refuse or discontinue participation at any time and the chance to ask anything about the study were given. All personnel information were kept entirely confidential and assured throughout the study period. Anyone was not forced to participate, as it was solely voluntary-based. The collected data was handled and secured by the principal investigator. The participants were informed that the data would be used only for research purposes. During data collection, any officer who exhibits symptoms of post-traumatic stress disorder or other related issues was consulted with or referred to a nearby hospital for professional help or treatment.

## Results

### Socio-demographic characteristics of participants

Data were obtained from 605 police officers, with a response rate of 95.4%. The mean age of the participants was found to be 33.96 ± 5.77, ranging from 20 to 52 years, and 380 (62.8%) of them were between 31 and 40 years old. The majority of the police officers were male (465, 76.9%). Most of the respondents in this study were married (287, 47.4%). Regarding educational status, 257 (42.5%) had attained a high school education. More than half (56.7%) of the police officers were junior officers. Out of the respondents, 227 (37.5%) had service experience of more than 5 years in the police force (**Table 1**).

**Table 1:** Socio-demographic characteristics of participants among police officers in central Gondar Zone North, (*n* = 605).

Variables	Categories	Frequency (*n* = 605)	Percent (100%)
Sex	Male	465	76.86
	Female	140	23.14
Age	20–30	140	23.14
	31–40	380	62.81
	41–50	75	12.40
	> 50	10	1.65
Rank	Lower	343	56.69
	Middle	211	34.88
	Top	51	8.43
Years of service	0–4	195	32.23
	5–10	227	37.52
	11–20	129	21.32
	> 20	54	8.93
Level of education	Primary	210	34.71
	Secondary	257	42.48
	College and university	138	22.81
Marital status	Single	247	40.83
	Married	287	47.44
	Divorced	49	8.10
	Widowed	22	3.64
Living arrangement	With family	261	43.14
	Alone	337	55.70
	Others	7	1.16

Other, includes (neighbors, child, and other relatives like sister and brother).

### Clinical and substance-related characteristics of the respondents

Out of the total participants, 144 (23.8%) had a family history of mental illness, whereas 68 (11.2%) had a history of mental illness themselves; 77 (12.7%) and 42 (6.9%) had lifetime suicidal ideation and attempts, respectively, and 111 (18.4%) police officers have reported known chronic medical conditions of different types. Moreover, out of 111 chronic medical illnesses, 40 (6.6%) had hypertension, 29 (4.8%) had asthma, 38 (6.3%) had diabetes mellitus, and four (0.7%) had cardiac problems. Regarding ever and current use of substance, 191 (31.6%) of the respondents were drinking alcohol at least once in their lifetime, whereas the khat and cigarette lifetime users were 28 (4.6%) and 10 (1.7%), respectively. Currently, 143 (23.5%), 14 (2.3%), and eight (1.3%), were using alcohol, tobacco, and khat, respectively (**Table 2**).

**Table 2:** Clinical characteristics of participants among police officers in Central Gondar Zone, North West, Ethiopia (***n*** = 605).

Variables	Categories	Frequency	Percent
Having known chronic medical illness	Yes	111	18.35
	No	494	81.65
Having known mental illness	Yes	68	11.24
	No	537	88.76
Lifetime suicidal ideation	Yes	77	12.73
	No	528	87.27
12-month suicidal ideation	Yes	32	5.29
	No	573	94.71
Lifetime suicidal attempt	Yes	42	6.94
	No	563	93.06
12-month suicidal attempt	Yes	8	1.32
	No	597	98.68
Family history of mental illness	Yes	144	23.80
	No	461	76.20
Ever substance use	Alcohol	191	31.57
	Khat	28	4.62
	Cigarette	10	1.65
Current substance use	Alcohol	143	23.64
	Cigarette	14	2.31
	Khat	8	1.32

### Psychosocial characteristics and levels of traumatic exposure

Regarding social support, the majority of the respondents (356, 58.8%) had poor social support, whereas police officers who had moderate and strong social supports were 182 (30.1%) and 67 (11.1%), respectively.

Concerning the level of exposure to a traumatic event, more than half of the police officers (369, 61%) have witnessed while others were brutally harassed or killed, and 236 (39%) were themselves exposed directly to traumatic events.

### Prevalence and associated factors of post-traumatic stress disorder

In this study, the overall prevalence of post-traumatic stress disorder among police officers was found to be 92 (15.2%), with 95% CI 12.5–18.3%.

Female sex, current alcohol use, years of service, marital status, 12-month suicidal ideation, police rank, chronic medical illness, history of mental illness, social support, and level of traumatic exposure were significantly associated factors with post-traumatic stress disorder in binary logistic regression at a *P*-value less than 0.2. Then, these variables were entered into the multivariable logistic regression model to control the confounding effects between the variables. Finally, multivariate analysis revealed that being female, current alcohol use, being directly exposed to traumatic events, and having poor social support were found to be significantly associated with PTSD, with a 95% CI at a *p*-value less than 0.05 (**Table 3**).

**Table 3:** Bivariable and multivariable analysis of factors associated with PTSD among police officers in Central Gondar Zone, North West, Ethiopia (***n*** = 605).

		PTSD				
Variables	Category	Yes	No	COR and 95% CI	AOR and 95% CI	*p*-value
Sex	Male	53	412	1	1	1
	Female	39	101	3.00 (1.88–4.78)	3.36 (1.95–5.78)	0.000***
Year of service	0–4	26	169	1	1	1
	5–10	29	198	0.95 (0.53–1.67)	1.00 (0.52–1.93)	0.987
	11–20	28	101	1.80 (1.00–3.24)	1.97 (0.98–3.93)	0.054
	> 20	9	45	1.30 (0.56–2.97)	1.55 (0.61–3.95)	0.356
Police rank	Lower	47	296	1	1	1
	Middle	31	180	1.08 (0.66–1.77)	1.08 (0.61–1.88)	0.785
	Top	14	37	2.38 (1.19–4.74)	1.93 (0.86–4.31)	0.106
Material status	Married	42	245	1	1	1
	Single	35	212	0.96 (0.59–1.56)	1.23 (0.70–2.16)	0.456
	Divorced	7	42	0.97(0.40–2.30)	1.21 (0.46–3.15)	0.688
	Widowed	8	14	3.33 (1.31–8.43)	1.87 (0.61–5.71)	0.271
Current alcohol use	No	60	402	1	1	1
	Yes	32	111	1.93 (1.19–3.11)	2.90 (1.64–5.12)	0.000***
Current suicidal thought	No	83	490	1	1	1
	Yes	9	23	(1.03–5.16)	1.96 (0.71–5.47)	0.192
History of mental illness	No	79	474	1	1	1
	Yes	13	39	1.99 (1.02–3.91)	1.39 (0.62–3.11)	0.421
History of medical illness	No	68	426	1	1	1
	Yes	24	87	1.72 (1.02–2.90)	1.83 (0.99–3.37)	0.051
Level of exposure	Being witnessed	45	324	1	1	1
	Directly involved	47	189	1.79 (1.14–2.79)	2.01 (1.16–3.48)	0.012*
Social support	Strong social support	6	59	1	1	1
	Moderate social support	15	188	0.78 (0.29–2.11)	1.06 (0.36–3.14)	0.903
	Poor social support	71	266	2.62 (1.08–6.32)	4.23 (1.58–11.36)	0.004**

*p-value ≤ 0.05; **p-value ≤ 0.01; ***p-value ≤ 0.001; 1, reference.

Hosmer and Lemeshow test = 0.82.

Female police officers were 3.4 times more likely to develop PTSD compared with male police officers (AOR = 3.36, 95% CI 1.95–5.78), and police officers who had a history of current alcohol use were about three times (AOR = 2.90, 95% CI 1.65–5.12) more likely to develop PTSD compared with those police officers who had no history of current alcohol use. Another associated factor was that those who were directly exposed to the traumatic event themselves were two times more likely to experience PTSD compared with those who witnessed while others were brutally killed or harassed (AOR = 2.01, 95% CI 1.16–3.48). Police officers who had poor social support were about 4.2 times more likely to develop post-traumatic stress disorder when compared to those who had strong social support (AOR = 4.25, 95% CI 1.58–11.36) (**Table 3**).

## Discussion

Post-traumatic stress disorder is one of the psychiatric disorders that follow after an exposure to a traumatic or stressful life event. Police officers are exposed to a number of traumatic events that put them at risk of developing post-traumatic stress disorder, which can affect their mental health and hinder their capacity to carry out their duties. In this study, the prevalence of post-traumatic stress disorder and its association with different factors were assessed.

The finding of the current study showed that the prevalence of post-traumatic stress disorder among police officers in the Central Gondar Zone was found to be 15.2% (95% CI: 12.5, 18.3%), which was in line with the findings of other studies conducted in the United Kingdom (UK), New York, and the USA, which reported 13%, 12.9%, and 13%, respectively (24–26).

The prevalence of PTSD in this study was higher than the studies done among Ugandan police officers, where the rate of PTSD was 7.4%, and Dutch police officers, who had a rate of PTSD of 7% (11, 27). Similarly, the post-traumatic stress disorder prevalence in the current study is also higher than other studies undertaken in Belgium (5.8%), Brazil (7.6%), and Canada (8.9%) (28–30). The possible reasons for the variation may be due to measurement tools and study design. The previous studies used retrospective, survey, and prospective study designs and the DSM-IV screening tool, whereas the current study used a cross-sectional study design and the DSM-V screening tool. Another possible explanation for the observed differences could be the differences in sociocultural background, socioeconomic level, and availability of healthcare facilities between those countries and Ethiopia. People living in low-socioeconomic countries like Ethiopia could have poor healthcare infrastructure and a shortage of trained health staff, which could deliver inadequate healthcare services. As such, post-traumatic stress disorder might not be identified and treated early (6).

However, the prevalence of post-traumatic stress disorder in this study was much lower than those reported in other African countries, particularly in South Africa, where a retrospective study undertaken among riot police officers in Cape Town and another among black police officers in Soweto and Pretoria reported a 36% and 41% rate of PTSD, respectively (31). The results of the current study were also lower than the study done among New Orleans police officers, which reported a 19% prevalence rate of PTSD (32). Another study carried out in South Korea also revealed a higher rate of PTSD of 41.11% (33). The discrepancy in the instruments and study design could be the cause of this variation. The previous studies used a different screeening tool to assess PTSD among police officers in South African context and used DSM-IV (31). Another possible cause of this variation might be the sample size difference between the current and previous studies. The previous studies used a large sample size, which is more than twice that of the current study.

Regarding factors affecting post-traumatic stress disorder, in this study, being female was found to be significantly associated with higher rates of post-traumatic stress disorder compared with male counterparts, which was 3.3 times more prevalent among female police officers than male officers. This finding, supported by other studies on Buffalo police officers and New York police officers, reported that PTSD was more prevalent in female police officers than in male police officers (34, 35). Other meta-analyses suggest that women are two to 3.5 times more likely than men to develop PTSD (36). The reason might be due to the fact that women are at a higher risk for potentially traumatic events like sexual assault, child sexual abuse, and attempted rape (37), and they are more likely to experience a lower threshold from exposure to psycho-trauma (38). Another possible reason could be that women tend to use more emotion-focused strategies, including rumination, as well as more avoidant coping strategies than men, which increases the probability of getting PTSD (39).

The result of the present study revealed that the level of traumatic exposure was another factor significantly associated with post-traumatic stress disorder among police officers. Indirect exposure to a traumatic event twice increased the odds of having PTSD. This finding is supported by other studies conducted in the United Kingdom and New York (24, 35). The possible reason could be that numerous behavioral and biological reactions are brought on by trauma exposure, and these responses interact with a person’s biology and genetics (40).

The present study also showed that post-traumatic stress disorder was significantly associated with current alcohol consumption. The odds of having PTSD were three times higher among police officers with a history of current alcohol consumption compared with their counterparts. This finding is supported by studies done in Uganda, Nigeria, and the UK, respectively (11, 41, 42). There are several possible reasons why police officers with high alcohol consumption may experience high levels of PTSD. Because alcohol use is linked to violence and accidents (43), particular PTSD symptoms such as avoidance and hyperarousal are more strongly linked to substance use (44), and frequent use of alcohol might play a more significant role in an attempt to escape from traumatic events and to cope with stress, pain, boredom, loneliness, and lack of other recreational activities (45).

Another contributing factor to the higher likelihood of developing PTSD is having poor social support, which leads to four times more likely having PTSD compared with those who have strong social support. This finding was supported by previous studies conducted in New York, Canada, and the Dutch, respectively (27, 28, 35). This result is also supported by another study conducted among New Zealand police officers, which revealed that being unable to discuss unpleasant events with coworkers increased the symptoms of post-traumatic stress disorder (PTSD) (46). This is due to the fact that social support plays a healing role after traumatic experiences. People who do not have strong connections with family or friends are more likely to have stronger physical and emotional reactions to trauma. As a result, trauma survivors who lack social support are at a significant risk of developing PTSD (47).

Our recommendation is to refer the Amhara Police Commission in a collaborative work which is needed for the Amhara Regional Health Bureau to extend mental health services to all police officers. In addition, our recommendation is forwarded to researchers as it is better to conduct a longitudinal research to identify the cause-and-effect relationship between PTSD and different factors.

## Limitations of the study

Due to the cross-sectional nature of the study design, we were not able to show the direction of causality between PTSD and the associated factors. Another limitation of this study was that it was also prone to reporting bias since the data was collected using self-administrative techniques. In addition, none of the above-mentioned tools was validated for this research; rather, we adapted these tools, and also there are data used in Ethiopia context.

## Conclusion

According to this study, police officers suffer from a substantial burden of post-traumatic stress disorder. Female sex, police personnel directly exposed to traumatic events, current alcohol users, and those who had poor social support were found to be strongly associated with post-traumatic stress disorder. Therefore, there is a need to establish a service to screen for and treat PTSD among police officers in Ethiopia.

## Data Availability

The original contributions presented in the study are included in the article/supplementary material. Further inquiries can be directed to the corresponding author.
